# Towards a Yersinia pestis lipid A recreated in an Escherichia coli scaffold genome

**DOI:** 10.1099/acmi.0.000723.v3

**Published:** 2024-07-17

**Authors:** Nathan D. McDonald, Erin E. Antoshak

**Affiliations:** 1 United States Army Combat Capabilities Development Command Chemical Biological Center, 8908 Guard St. E3831, Gunpowder, MD 21010, USA; 2 Excet Inc. 6225 Brandon Ave #360, Springfield, VA 22150, USA

**Keywords:** CRISPR-Cas9, genome engineering, lipid A, lipopolysaccharide

## Abstract

Synthetic biology and genome engineering capabilities have facilitated the utilization of bacteria for a myriad of applications, ranging from medical treatments to biomanufacturing of complex molecules. The bacterial outer membrane, specifically the lipopolysaccharide (LPS), plays an integral role in the physiology, pathogenesis, and serves as a main target of existing detection assays for Gram-negative bacteria. Here we use CRISPR/Cas9 recombineering to insert *Yersinia pestis* lipid A biosynthesis genes into the genome of an *Escherichia coli* strain expressing the lipid IV_a_ subunit. We successfully inserted three genes: *kdsD*, *lpxM*, and *lpxP* into the *E. coli* genome and demonstrated their expression via reverse transcription PCR (RT-PCR). Despite observing expression of these genes, analytical characterization of the engineered strain’s lipid A structure via MALDI-TOF mass spectrometry indicated that the *Y. pestis* lipid A was not recapitulated in the *E. coli* background. As synthetic biology and genome engineering technologies advance, novel applications and utilities for the detection and treatments of dangerous pathogens like *Yersinia pestis* will continue to be developed.

## Data Summary

All supporting data have been provided within the article or through supplementary data files.

## Introduction

**Fig. 1.**
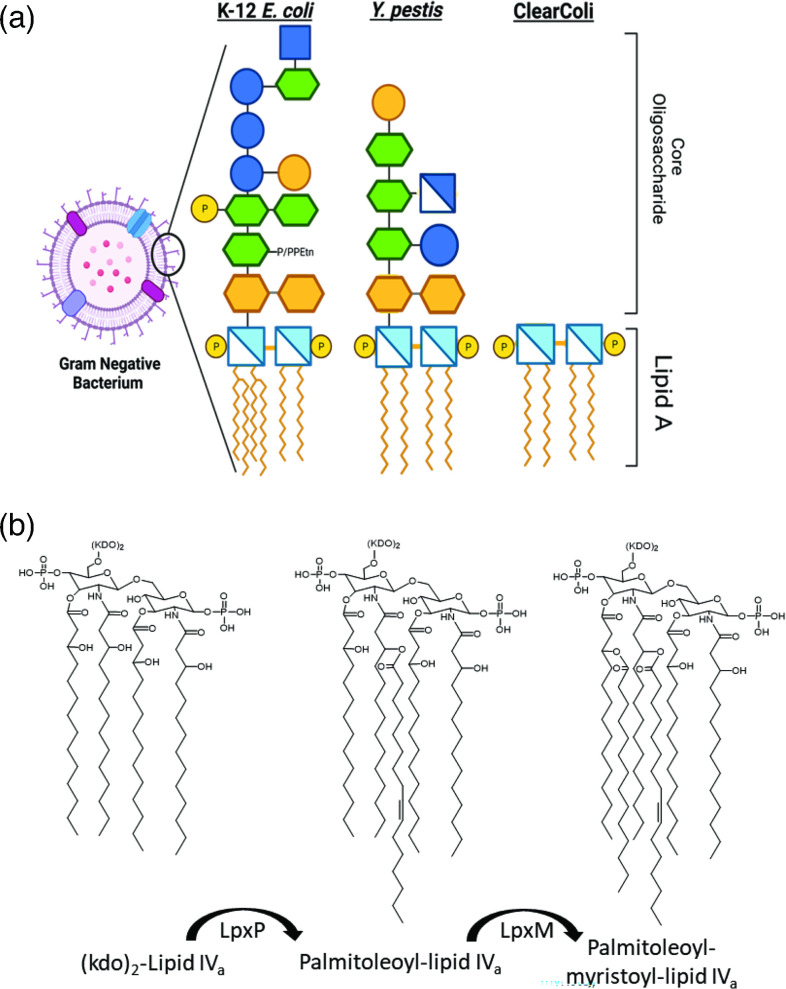
Representative bacterial outer-membrane structures and late acyl transferases biosynthesis. (**a**) Graphical representation of lipooligosaccharide structures from *E. coli* K-12, *Yersinia pestis* and the mutant *E. coli* strain ClearColi. Individual symbols are indicated as follows: light blue crossed square – glucosamine; orange hexagon – 3-Deoxy-d-manno-Oct-2-ulosonic acid (Kdo); flat hexagons – Heptoses; blue circle – glucose; orange circle – mannose; dark blue crossed square – glucosamine; As indicated the lipooligosaccharide is comprised of two main domains, the core oligosaccharide, which is generally conserved within a species and the lipid A, which forms the outer leaflet of the lipopolysaccharide. (**b**) The biosynthetic pathway and function of late acyl transferases LpxP and LpxM. The acyl chains are added successively following the formation of lipid IV_a_ and addition of the two KDO moieties.

A hallmark defining feature of Gram-negative bacteria is the presence of an outer membrane primarily comprised of either lipopolysaccharide (LPS) or lipooligosaccharide (LOS). As the names imply, the LPS and LOS macromolecules contain both a lipid region and carbohydrate chains. The outer-membrane molecules can generally be divided into three distinct domains: the lipid A, core oligosaccharide, which together make up the LOS (Fig. 1a) and finally the repeating carbohydrate O-antigen completing the LPS [1]. Each of the domains have specific and unique structures and properties which contribute to a myriad of phenotypes for individual bacteria. While there are exceptions, one lipid A unit is comprised of linked d-glucosamine disaccharide backbone, which is phosphorylated at positions 1′ and 4′ of the carbohydrates [1,3]. The backbone is acylated with branching fatty acid chains of varying lengths and substitutions depending on the species. This main unit of lipid A can be further modified by various additions including phosphates, carbohydrates, and other small molecules, which can alter the overall charge [1,3]. Together, it is the lipid A unit which is responsible for the endotoxic properties of LPS by activation of the innate immune system via recognition by toll-like receptor TLR4 [2].

*Yersinia pestis*, the causative agent of the plague, possesses a unique lipid A that undergoes a variety of changes as the organism transitions through lifecycle and infection stages. The *Y. pestis* lipid A is typical in that it contains a glucosamine carbohydrate backbone like other enterobacteria. Branching from the linked glucosamine residues are a variety of fatty acid acyl chains, the structure and composition of which are dependent on the temperature at which the organism is growing (Fig. S1, available in the online Supplementary Material) [4,6]. At 37 °C, the temperature encountered during infection, the *Y. pestis* lipid A is tetra-acylated with hydroxymyristate (Fig. S1) [6,7]. This tetra-acylated lipid A is known as lipid IV_a_ and is considered the minimal lipid unit required for bacterial survival under laboratory conditions [8]. Furthermore, this lipid IV_a_ has been shown to be less immunogenic during *Y. pestis* infection, which contributes to the overall pathogenesis during infection [4,7,9, 10]. At lower temperatures, 20–28 °C, the *Y. pestis* lipid A is hexa-acylated with the addition of a palmitoleate residue, catalysed by the enzyme LpxP, and a myristate group catalysed by the enzyme LpxM (Figs 1b and S2) [5,9, 10].

Because of the immunostimulatory nature of the outer membrane of bacteria, it is well established that the lipopolysaccharide and outer-membrane proteins are the primary targets of therapeutic antibodies. This is particularly true for *Yersinia pestis* in that many therapeutic antibodies exist that target the F1 protein, which is responsible for the capsule-like antigen present during infection [11,17]. However, studies have demonstrated that highly virulent F1-negative strains of *Yersinia pestis* exist, which limits the efficacy of F1-specific antibodies and detection methodologies [18,19]. Furthermore, because of the nature of *Yersinia pestis* and its classification as a potential biothreat agent, the research and development activities involved with the development of novel therapeutics and detection capabilities is hampered or limited to utilizing attenuated strains of the organism. Previous studies have demonstrated that outer-membrane vesicles can be engineered and utilized to develop novel therapeutics [20,22]. We hypothesized that if specific epitopes from the *Yersinia pestis* outer membrane could be engineered and expressed in lab strains of * E. coli*, future work could leverage this engineered strain for the development of novel therapeutics and detection assays for *Y. pestis*.

Here we set out to utilize CRISPR/Cas9 recombineering technologies to genetically engineer a commercially available, mutated strain of *E. coli* that expresses lipid IV_A_ as its outer-membrane unit [8]. Using synthetic DNA, we inserted three *Y. pestis* genes involved in lipid A biosynthesis into this *E. coli* scaffold to determine if we could reconstitute the *Y. pestis* lipid A in this strain.

## Methods

### Growth conditions

Organisms and plasmids used in this work are listed in Table 1. The endotoxin-free *E. coli* (DE3) ClearColi electrocompetent cells were purchased through Lucigen. The two plasmid CRISPR-Cas9 system utilized includes a pEcCas (plasmid no. 73 227) and guide pEcgRNA plasmid (plasmid no. 166 581) that are available through Addgene and previously described [23]. The kdsD inducible expression vector was purchased from ATUM Inc. and the sequence is available in the Supplementary Material. All strains were grown in Luria–Bertani-(LB) Broth (Miller) (Sigma-Aldrich; St. Louis, MO). Antibiotics were used as needed at the following concentrations: kanamycin (kan) (50 µg ml^−1^), spectinomycin (spec) (50 µg ml^−1^), ampicillin (amp) (100 µg ml^−1^). All routine culturing was performed at 37 °C with shaking (250 r.p.m.).

**Table 1.** Strains and plasmids used in this study

Strain or plasmid	Genotype or phenotype	Reference or source
pEcCas	pWM119: Cas9, sgRNA (amp->kan)	Addgene no. 73227[23]
pEcgRNA	pTargetF: *ccdB*, Spectinomycin	Addgene no. 166581[23]
pEcgRNA-N20-*lpxM*	Guide RNA vector targeting *lpxM* insertion site in ClearColi genome	This study
pEcgRNA-N20-*lpxP*	Guide RNA vector targeting *lpxP* insertion site in Clearcoli genome	This study
pEcgRNA-N20-*kdsD*	Guide RNA vector targeting *kdsD* insertion site in Clearcoli genome	This study
*Escherichia coli* ClearColiBL21(DE3)	F^-^ ompT hsdS_B_ (r_B_^-^ m_B_^-^) gal dcm lon λ(DE3 [lacI lacUV5-T7 gene 1 ind1 sam7 nin5]) msbA148 Δ*gutQ* Δ*kdsD* Δ*lpxL* Δ*lpxM* Δ*pagP* Δ*lpxP* Δ*eptA*	Invitrogen
*Escherichia coli*BL21 (DE3)	fhuA2 [lon] ompT gal (λ sBamHIo ∆EcoRI-B int::(lacI::PlacUV5::T7 gene1) i21 ∆nin5) [dcm] ∆hsdS	New England BioLabs
ClearColi:*lpxM*	Clearcoli genome with *Y. pestis lpxM* inserted in the cognate genomic loci	This study
ClearColi:*lpxM:lpxP*	Clearcoli:*lpxM* genome with *Y. pestis lpxP* inserted in the cognate genomic loci	This study
ClearColi:*lpxM:lpxP:kdsD*	Clearcoli:*lpxM:lpxP* genome with *Y. pestis kdsD* inserted in the *gutQ* genomic loci	This study
ClearColi:*lpxM:lpxP:kdsD*	Clearcoli:lpxM:lpxP:kdsD +an inducible expression vector containing the *Y. pestis kdsD* gene. Kan^r^	This study

### Generating repair DNA templates

The donor DNA was supplied as gBlocks (Integrated DNA Technologies; Coralville, IA). The gBlock was constructed to include the specific gene of interest while also including the N20 sequence and PAM site indicated in the ClearColi sequence. Flanking the gene are ~500 bp regions homologous to the target insertion site in the Clearcoli genome. The gBlock is used as a template in a PCR amplification reaction. The reaction was performed with Phusion High Fidelity DNA Polymerase (New England Biolabs; Ipswich, MA) following the provided NEB Phusion protocol and utilizing the gBlock primers listed in Table 2. For confirmation, gel electrophoresis was performed of the amplified gBlock at 120 V for 30 min on a 1 % agarose gel stained with SYBR Safe DNA Gel Stain. The amplified DNA was purified using a DNA Clean and Concentrator-100 kit (Zymo Research; Irvine, CA).

**Table 2.** Primers used in this study

Primer ID	Primer sequence
5EC*lpxM*ctrl	ATT AAT TAA CAT CCA TTC GCA GCC G
3EC*lpxM*ctrl	CCT ACA GTT CAA TGA TAG TTC AAC AGA TTT CG
Yp_*LpxM*.F	TCG GTT TCA CCC TCT TTC CG
Yp_*LpxM*.R	ATT AGC TGG CAT AGG GCG TC
*LpxM*_Seq_F	GAA GCG GTT AAT CTG CTG CG
*LpxM*_Seq_R	GGA TAA ACC AGC AGG CCG TA
*LpxM*_gblock_f	GTG CAC CGG CGT AAC GCC ACT CAAAAA AAG CAC CGA CTC G
*LpxM*_gblock_r	TCA TGG TCG CAG CTA CAC CA
pTargetF-N20R	ACT AGT ATT ATA CCT AGG ACT GAG
5EClpxPctrl	AGT AGC TGA AAG CAG TCA GC
3EClpxPctrl	AGT AAC TTA CAA GTG TCT CAT ATC GG
Yp_*LpxP*.F	TAC AGC GAA GGT TCG CCA AT
Yp_*LpxP*.R	ACG CGC ACA ACA AGG TAA AC
*LpxP*_Seq_F	TGC AAG ACT GTT GTG TAC GGA
*LpxP*_Seq_R	GTA TTT TAC CGT GGG CAT CAC C
Kdsd NEW pecgrna n20 F	CAGTCCTAGGTATAATACTAGTCGGAATAGGAACTAAGGAGGGTTTTAGAGCTAGAAATAG
Yp_kdsD_screen_F	GAGTCACGCCATATCACTGC
CC_gutQ_screen_rev	GTGGCGGAAAGTGAGTTGTT
lpxM _Bl21_Screen_F	CGTATCAGCTCTGGTCTGCC
lpxM_Yp_Screen_R	CGCGACCTATAAGGCGACAT
lpxP_BL21_Screen_F	ATGAGTGCAGCGAGGATCAC
lpxP_Yp_Screen_R	AATTAGTGCAGACCTGGGGC
cysG_F	TTGTCGGCGGTGGTGATGTC
cysG_R	ATGCGGTGAACTGTGGAATAA

### Guide plasmid construction

The pEcgRNA guide plasmid was constructed to incorporate the targeting sgRNA by performing inverse PCR with overlapping primers. One primer is specific for each insertion and contains the 20-basepair gRNA target sequence listed in Table 2. The guide plasmid inverse PCR is followed by Gibson Assembly with NEBuilder HiFi DNA Assembly Master Mix. Confirmation of the incorporation of the N20 guide region in the plasmid is noted by successful transformation into NEB 5-alpha Competent *E. coli*. The N20 sequence replaces the selective marker *ccdB* gene. Lastly the plasmid is isolated by the ZymoPURE Plasmid Miniprep Kit from Zymo Research.

### Genome editing with CRISPR/Cas9

First the pEcCas plasmid is electroporated into the ClearColi cells in electroporation cuvettes (0.1 cm gap) and pulsed at 1.8 kV. Positive colonies were selected on LB +kan plates. ClearColi and subsequent mutant strains containing pEcCas plasmid were made electrocompetent to incorporate the specific pEcgRNA plasmid and donor DNA. The gene editing was conducted as previously described [23,24]. In brief, the electrocompetent cells were transformed as described above with 100 ng of the specific pEcgRNA and 400 ng of the linear repair template and clones were selected on LB +Kan+ Spec. The successful insertion of each gene of interest was confirmed by colony PCR utilizing the screening primers listed in Table 2.

### Plasmid curing

After confirmation of gene insertion the pEcgRNA plasmid was cured as previously described [23,24]. In brief, cells are inoculated in an overnight culture in LB containing kanamycin and 100 mM rhamnose at 37 °C with shaking. The cultures were plated onto LB-kan plates and incubated overnight. Selected colonies were then added to 50 µl LB+kan and streaked onto LB+kan/spec plates. After overnight incubation, plates that have no growth observed on the LB+kan/spec plates are considered cured of the pEcgRNA plasmid. For curing of pEcCAs, colonies that were cured of pEcgRNA were incubated overnight in LB and 100 µl spread on LB plates containing 10 % w/v sucrose and incubated overnight. From the sucrose plates, colonies were selected and passaged to LB plates +/-kan. Clones sensitive to kan were cured of pEcCas.

### Reverse transcription-PCR expression analyses

The Qiagen Rneasy Mini Kit was utilized to isolate RNA from each strain. Overnight cultures were prepared of the strains and the following day 200 µl of each culture is added to new media and grown to 1×10^7^ cells and centrifuged at 5000 ***g*** for 5 min at 4 °C. The RNA was then isolated according following the manufacturer’s protocol. cDNA synthesis was performed using SuperScript IV Reverse Transcriptase kit with 50 µM random hexamers following the recommended protocol. Finally, a PCR reaction with Phusion High Fidelity DNA Polymerase is performed on the reverse transcription-PCR samples using primers specific to the * Y. pestis* genes (Table 2). Amplified DNA was visualized on a 1 % agarose SYBR Safe stained gel is run of the PCR samples at 120 V for 30 min.

### MALDI-TOF mass spectrometry

Preparation of lipid A extracts for MALDI-TOF analyses were prepared as previously described [25]. In brief, overnight cultures were prepared in 5 ml LB–Miller broth with their respective selective antibiotic at 37 °C and shaking overnight. Next 1 ml of overnight culture is taken from each sample and resuspended in 400 µl of 100 mM sodium acetate pH 4.0. After resuspension the samples were incubated at 100 °C for 30 min, while being vortexed every 10 min. After cooling on ice, the samples were centrifuged at 8000 ***g*** for 5 min. The supernatant was removed, and the cell pellets were washed with 95 % ethanol and 100 µl of 12 : 6 : 1 chloroform/methanol/water was added, and the samples were centrifuged at 5000 ***g*** for 5 min. The supernatant spotted on stainless steel plates previously spotted with 10 mg ml^−1^ norharmane in 12 : 6 : 1 chloroform/methanol/water. Mass spectra were collected under negative ion mode on the smartfleX MALDI-TOF MS System (Bruker).

## Results

### Genetic insertions of *Yersinia pestis* lipid A biosynthesis genes into *E. coli* genome

Previously, Mamat *et al*. utilized a series of genetic deletions and mutations to generate ‘endotoxin free’ strains of *E. coli* in various backgrounds, including *E. coli* BL21(DE3) [8]. The resulting strains retained the minimal lipid A unit required for survival, lipid IV_a_, and were created as ideal strains for the expression and purification of proteins which typically bind endotoxin (Fig. 1a) [8]. Here we chose this strain, known as ClearColi, as a scaffold in which we can attempt to recreate the wild-type *Yersinia pestis* lipid A in an *E. coli* genetic background. Utilizing several previously described reports which have detailed the structure and genetic biosynthesis pathways associated with outer-membrane biosynthesis in *Y. pestis*, we selected three genes to insert into the ClearColi genome [5,9, 10, 26]. Specifically, the genes *lpxM* and *lpxP* encoding late acyl-transferase enzymes, that catalyse the addition of myristate and palmitoleate, respectively, to the lipid chain (Fig. 1b). Previous reports have suggested that lipid IV_a_ is not the substrate for LpxM or LpxP and that the genes act downstream of kdo incorporation, requiring (kdo)_2_-lipid IV_a_ as a substrate [8,27], therefore we determined that 3-deoxy-d-manno-oct-2-ulosonic (kdo) carbohydrate biosynthesis would need to be restored through the addition of *kdsD*, which is a d-arabinose 5-phosphate isomerase.

**Fig. 2.**
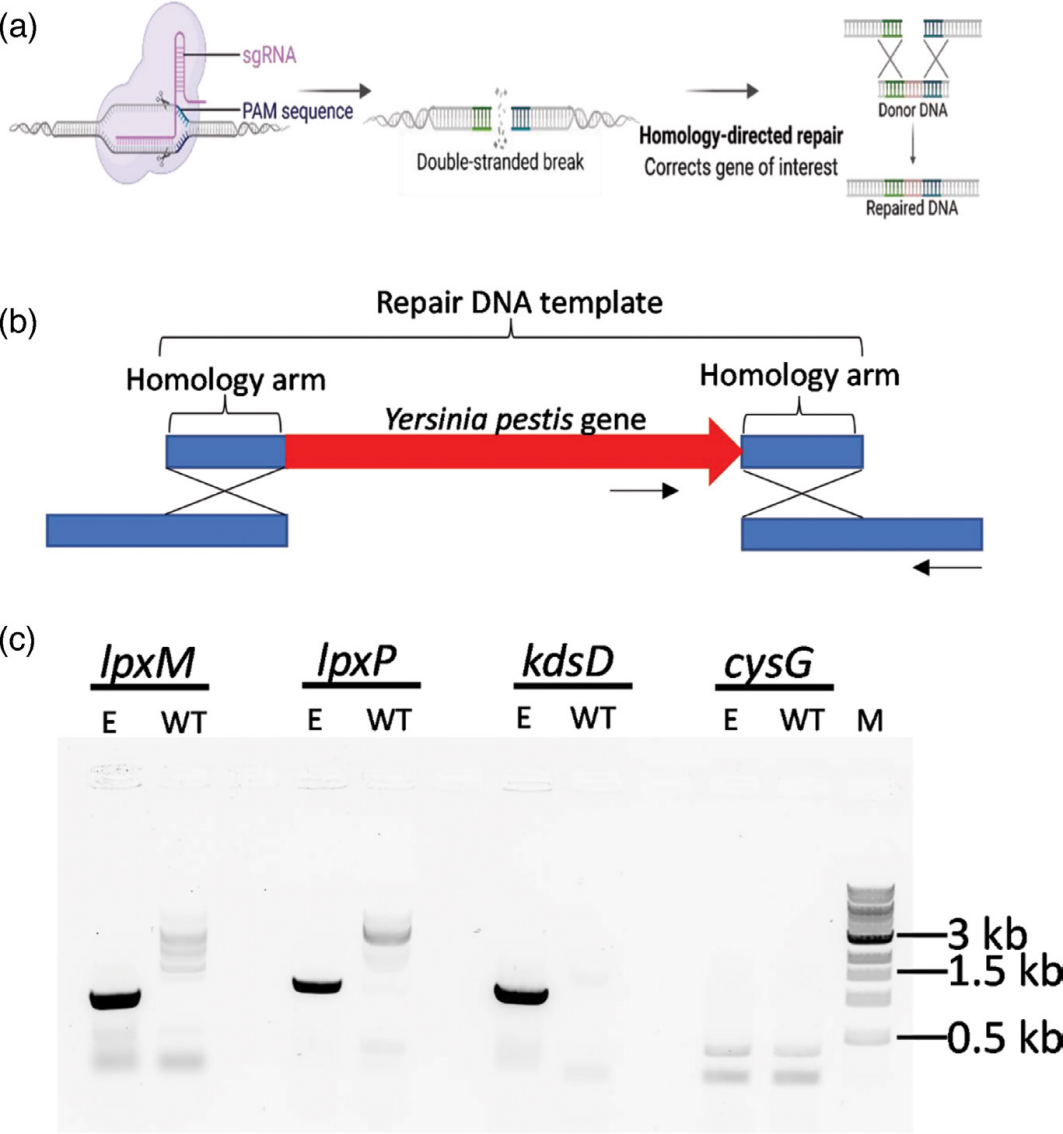
Insertion of *Y. pestis* genes into the ClearColi genome with CRISPR/Cas9 recombineering. (**a**) Schematic representing CRISPR/Cas9 site directed double-stranded DNA cleavage and homology guided repair. (**b**) Design of repair templates utilized for genome editing. The *Yersinia pestis* genes of interest (red) were designed to have regions of homology to the ClearColi genome (blue). Primers for confirming the insertion are designated by the black arrows. (**c**) 1 % SYBR Safe stained agarose gel includes amplified DNA from a colony PCR screen. The letter E designates the Clearcoli:lpxM:lpxP:kdsD edited strain and the WT designates the wildtype Clearcoli template. The amplified reactions from the internal *Y. pestis lpxM* primer and primer outside of the *E. coli* ClearColi homology arm region with an expected size of 861 bp. Similarly explained primer set for the specific *Y. pestis lpxP* insertion with an expected size of 1 019 bp. The specific *Y. pestis kdsD* insertion with an expected size of 967 bp. Finally, an internal housekeeping control *csyG* with an expected size of 105 bp. The molecular weight marker is the NEB Quick-Load 1 kb DNA Ladder. Data is representative of at least three independent biological replicates.

Having identified the genes of interested from *Y. pestis* to insert in the ClearColi genome, we utilized a two-plasmid CRISPR/Cas9 genome-editing system designed for *E. coli* to make the insertions [23]. First, the ClearColi genome was sequenced at the respective homolog loci for *lpxM*, *lpxP* and *ksdD* to identify the flanking protospacer adjacent motif (PAM) site that would be the site of insertion for the *Y. pestis* genes following Cas9 cleavage and subsequent repair (Fig. 2a and File S1). We were unable to acquire sequencing data that aligned with previous reports of the *E. coli kdsD* deletion in the ClearColi background, which prevented us from identifying a PAM that could be used for targeting as an insertion site. The *E. coli* genome contains two homologous d-arabinose 5-phosphate isomerases, KdsD and GutQ, both of which have been shown to catalyse kdo biosynthesis [8,28, 29]. Therefore, we chose to insert the *Y. pestis kdsD* gene into paralogous *gutQ* loci in the ClearColi strain. Once the PAM was identified for each target, the repair template, which includes the *Y. pestis* gene of interest flanked by ~500 bp of ClearColi genome on either side was acquired as synthetic DNA and amplified by PCR (Fig. 2b). Next the Cas9 vector, the guide RNA targeting vector, and the repair template DNA were transformed into the ClearColi strain and resultant transformants were screened for successful gene insertion. As indicated by the black arrows in Fig. 2b, inserted *Y. pestis* genes were confirmed by PCR utilizing one primer within the *Y. pestis* gene and the other outside of the repair DNA template (Fig. 2c). In succession, we generated ClearColi:*lpxM*, ClearColi:*lpxM:lpxP*, and finally ClearColi:*lpxM:lpxP:kdsD* strain derivatives.

### *Yersinia pestis* lipid A biosynthesis genes are expressed in the engineered *E. coli* ClearColi strain

**Fig. 3.**
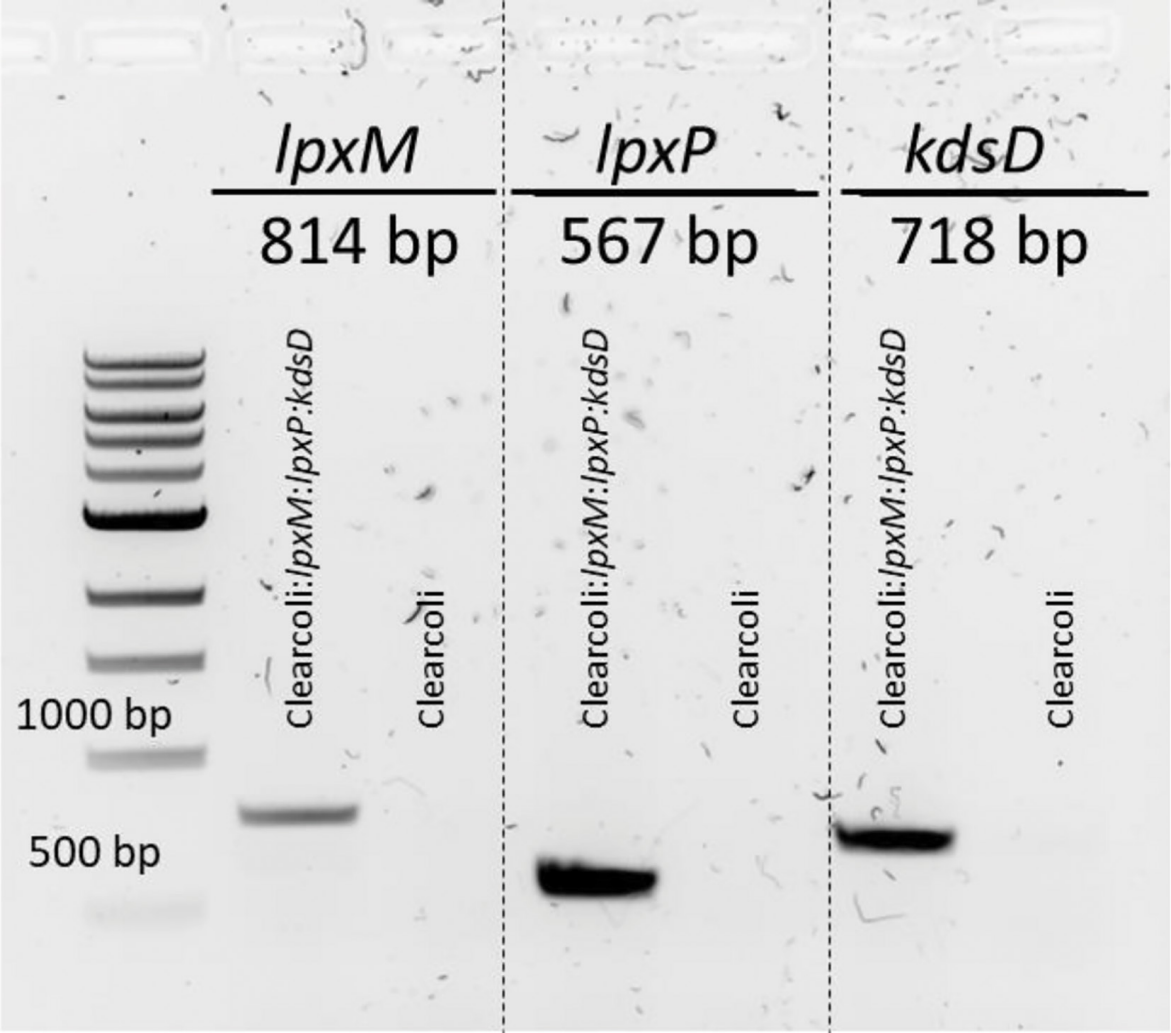
Reverse transcription-PCR expression analyses of *Y. pestis* genes inserted in the ClearColi genome. Total RNA was isolated from mid-exponential cultures to screen for *Y. pestis* gene expression. The reverse transcribed cDNA was used as templates for the PCR reactions. The products were analysed on a 1 % SYBR Safe stained agarose gel. cDNA from the WT ClearColi strain was used as a negative control for expression analyses. Data is representative of at least three independent biological replicates.

Having successfully confirmed the insertion of the three *Y. pestis* lipid A biosynthesis genes into the background ClearColi genome we next set to confirm that the genes were being expressed as we would expect. Through the genetic manipulations, only the coding regions of the *Y. pestis* genes were inserted into the ClearColi genome meaning that the transcription of the genes is under the control of the native *E. coli* promoters. Total RNA was isolated from early logarithmic phase growth for wild-type ClearColi and ClearColi:*lpxM:lpxP:kdsD* and cDNA synthesis was performed. The expression of *lpxM*, *lpxP*, and *kdsD* was confirmed using primers specific to the *Y. pestis* gene with the cDNA as a template and analysed by gel electrophoresis. As expected, we observed expression for each of the *Y. pestis* genes in the edited strain with no expression in the wild-type ClearColi (Fig. 3). Together this data demonstrates that not only have the *Y. pestis* genes been inserted into the ClearColi genome, but they are transcribed under the control of the native *E. coli* promoters.

### *Yersinia pestis* lipid A biosynthesis genes are unable to restore hexa-acylated lipid A in engineered *E. coli*

**Fig. 4.**
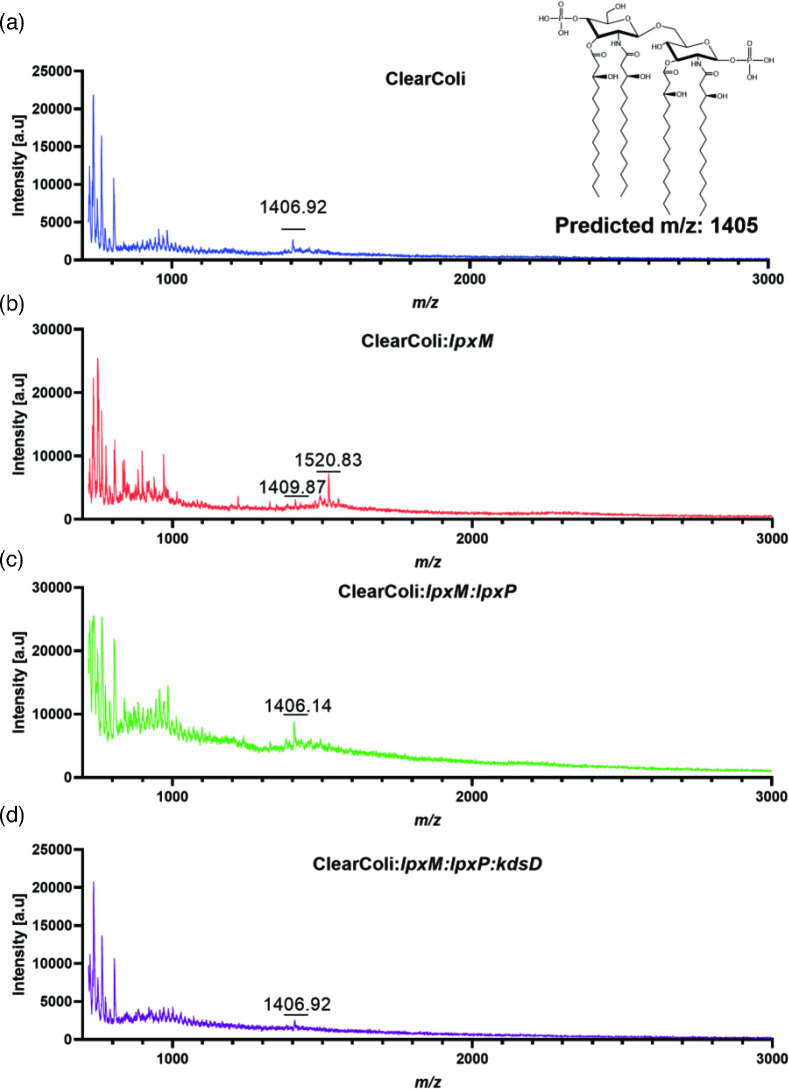
MALDI-TOF characterization of lipid A structures isolated from engineered ClearColi strains. Lipids were isolated from (a) WT ClearColi inset represents the expected lipid IV_a_ structure and predicted mass, (**b**) ClearColi:*lpxM*, (**c**) ClearColi:*lpxM:lpxP* and d) ClearColi:*lpxM:lpxP:kdsD*.

Having confirmed that each of the genes were being expressed as expected, we set out to determine if we have successfully restored the function of LpxM, LpxP, and KdsD in lipid A biosynthesis in the ClearColi background with *Y. pestis* genes. The lipids from wild-type ClearColi, ClearColi:*lpxM*, ClearColi:*lpxM:lpxP*, and ClearColi:*lpxM:lpxP:kdsD,* were prepared utilizing a previously described method for analyses by matrix-assisted laser desorption ionization – time of flight (MALDI-TOF) mass spectrometry [25]. In wild-type ClearColi, we observed a major peak at 1404 *m/z* corresponding to the presence of just lipid IV_a_ as reported previously (Fig. 4a) [8]. Next, we examined the lipid structures of ClearColi:*lpxM* and ClearColi:*lpxM:lpxP* to determine if the late acyl transferase genes were functional in this background. In either background, we only observed the 1404 *m/z* corresponding to the lipid IV_a_ rather than the expected mass of 1852 *m/z*, which would indicate that the enzymes are functioning as expected (Fig. 4b, c). This result is not surprising as mentioned above, lipid IV_a_ is not the substrate for LpxM or LpxP and that the genes act downstream of kdo incorporation, requiring (kdo)_2_-lipid IV_a_ as a substrate [8,27]. Finally, we characterized the lipids from ClearColi:*lpxM:lpxP:kdsD* by MALDI-TOF to determine if hexa-acylated lipid A biosynthesis was restored. Again, in this case, we only observed the 1404 *m/z* corresponding to lipid IV_a_ with no high molecular weight moieties identified (Fig. 4d). Because *Yersinia pestis* displays multiple lipid A structures depending on the growth temperature, we evaluated the ClearColi*:lpxM:lpxP:kdsD* strain grown at 28 °C, which in *Y. pestis* would result in the fully acylated phenotype. Under these growth conditions, the lipid IV_a_ of 1405.9 *m/z* was observed absent of acylation from LpxM or LpxP (Fig. S2). As we did not observe the acylation patterns, we speculated that the KdsD was not functioning as expected. To test this hypothesis, we tested the expression of *kdsD* from an inducible extrachromosomal plasmid. The complemented strain was grown at 26 and 37 °C under inducing conditions and the isolated lipid A was analysed. Again, no high molecular weight species that would be indicative of acylation were observed, just the corresponding lipid IV_a_ mass of 1405.9 *m/z* (Fig. S2). Taken together, these results indicate that simply adding the genes encoding LpxM, LpxP, and KdsD is not sufficient to rescue lipid A biosynthesis in the *E. coli* ClearColi background. Future work is required to determine what other genetic modifications need to occur to rescue the lipid A biosynthesis in this strain.

## Discussion

Here we set out to recapitulate *Y. pestis* lipid A biosynthesis in a previously engineered strain of *E. coli*, which expresses the tetra-acylated lipid IV_a_ outer-membrane structure. We hypothesized that if we inserted three critical genes; *kdsD*, *lpxM* and *lpxP* from *Y. pestis*, into *E. coli* ClearColi, then the engineered strain would express a hexa-acylated lipid A, like what is observed in * Y. pestis* at lower temperatures. Despite successfully inserting the genes into the homologous loci in *E. coli* ClearColi and confirming expression, we were unable to restore the hexa-acylated lipid A in the engineered strain as determined by MALDI-TOF analyses of isolated lipid A.

There are several potential explanations as to why hexa-acylated lipid a was not achieved in the engineered *E. coli*. As expected, we did not observe the acylation from insertion of LpxM and LpxP in the absence of kdo biosynthesis as lipid IV_a_ is not a substrate for these enzymes [8]. However, once the *Y. pestis kdsD* was inserted in the ClearColi genome we expected kdo biosynthesis to be restored and LpxM and LpxP to be functional, which was not observed. We utilized an expression vector to evaluate *Y. pestis kdsD* complementation and again did not observe the expected phenotype. Future work will determine whether the *Y. pestis* KdsD is sufficient to restore kdo biosynthesis in the *E. coli* background.

In addition to the potential non-functional kdo biosynthesis, a second explanation for the inability to restore hexa-acylated lipid A is through a point mutation in the gene encoding the lipid A transport protein MsbA. Previous studies have demonstrated that for deficient kdo biosynthesis to be a non-lethal mutation, suppressor mutations in *msbA* or *yhjD* are required under fast growing conditions [27]. In fact, the ClearColi strain does have a G to A mutation in *msbA* at position 52, which enables this strain to be viable with lipid IV_a_ as its outer membrane [8]. Furthermore, in the absence of these suppressor mutations, *E. coli* strains exhibiting just lipid IV_a_ or (kdo)_2_-lipid A have only been achieved under slow-growing medium conditions at lower temperatures not evaluated in this study [27]. Future efforts are targeted at exploring repairing the *msbA* mutation in the ClearColi strain or via overexpression of WT *msbA* in the engineered strain to determine if hexa-acylated lipid A can be restored.

Bacterial outer membranes are known to be highly immunostimulatory and these macromolecules can be harnessed for both immunotherapy development and targets for the detection and typing of pathogens. *Yersina pestis* is a dangerous pathogen and potential biological threat that can require medical intervention and detection systems. Current medical interventions include standard antibiotics, however, *Y. pestis* is known to become multi-drug resistant making the need for alternative treatments a priority. Similarly, current detection methodologies rely on the presence of the F1 proteinaceous capsule, however not all highly virulent strains of *Y. pestis* express the F1 capsule. The goal of this work was to utilize CRISPR/Cas9 genome engineering to recreate the *Y. pestis* outer membrane in an *E. coli* scaffold strain. If it had been successful, this engineered strain would be the beginning of a new tool that could be utilized as an antigen for development of novel therapeutics and detection assays for *Yersinia pestis*.

## Limitations and future directions

The purpose of this work was to begin developing a recombinant research tool that could be utilized for the development of novel therapeutics and detection devices for the human pathogen *Yersinia pestis*. Through this work we were unable to recreate aspects of the lipid A molecule in the ClearColi base strain as a scaffold. One of the primary limitations with this approach was utilizing the *E. coli* ClearColi scaffold as the strain selected for engineering. Because of the extensive genetic modifications that have occurred within this strain, we found it difficult to pinpoint the point-of-failure within the strain. Had we started with an * E. coli* strain expressing a fully functional LOS and carried out our genetic insertions in that background we would have known if a *Y. pestis* gene resulted in a phenotypic change more readily. In addition, the genome of the ClearColi strain has been sequenced but not available in a genome database. While it is an *E. coli* BL21 derivative, we identified scars present in the genome at deletion loci that were not anticipated. These scars may be impacting the regulation of our inserted *Y. pestis* genes depending on the location of the PAM at each insertion site.

Another limitation of this current study is that the transcriptional regulation of the *Y. pestis* genes are not accounted for within the scaffold *E. coli* strain. In this study, the coding sequence for each gene from *Y. pestis* was inserted into the cognate loci of *E. coli* with the intention that the native *E. coli* regulatory components would control the transcription of the gene. For future iterations of this work, it will be important to understand the impact of these regulatory regions to better generate the specific * Y. pestis* outer-membrane antigens of interest.

In future work, a more reasonable approach would be to utilize inducible expression vectors in the *E. coli* scaffold to ensure the desired phenotype prior to genome insertion via CRISPR/Cas9. This approach will be time saving and validate that the recombinant enzymes are functional in the derived strain. As described above, immediate next steps include testing the hypothesis that point mutations in the *msbA* gene are preventing the formation of lipid IV_a_, which has been previously described in other organisms. This hypothesis could be tested via overexpression of a wild-type *msbA* from an inducible vector followed by evaluation of the resultant lipid A via MALDI-TOF mass spectrometry. If this overexpression of *msbA* does in fact resolve lipid A formation, the point mutation within the genome can be corrected. Ultimately, once the desired lipid A structure is completed, within the scaffold *E. coli*, a series of additional genetic mutations would be made that would drive the synthesis of the *Y. pestis* core oligosaccharide, completing LOS in the *E. coli* background. From this point additional *Y. pestis* antigens such as F1 may be added to better simulate the key components of *Y. pestis* for the development of novel therapeutics and detection assays.

## supplementary material

10.1099/acmi.0.000723.v3 Uncited Supplementary Material 1.

## Abbreviations

KDO: 3-deoxy-d-manno-oct-2-ulosonic
LOS: lipooligosaccharide
LPS: lipopolysaccharide
PAM: protospacer adjacent motif
