# Supplementary material for: Towards a Yersinia pestis lipid A recreated in an Escherichia coli scaffold genome
Source: Access Microbiol. 2024 Jul 17;6(7):000723.v3. doi: 10.1099/acmi.0.000723.v3 (PMC11316592; doi:10.1099/acmi.0.000723.v3)
Supplement: Uncited Supplementary Material 1. [file acmi-6-00723-s001.pdf]

Towards a *Yersinia pestis* lipid A recreated in an *Escherichia coli* scaffold genome

Nathan D. McDonald[1‡] and Erin E. Antoshak[1, 2]

[1]United States Army Combat Capabilities Development Command-Chemical Biological Center, 8908 Guard St. E3831, Gunpowder, MD 21010

[2] Excet, Inc. 6225 Brandon Ave #360, Springfield, VA 22150

Corresponding Author:

Nathan D. McDonald[‡]

United States Army Combat Capabilities Development Command-Chemical Biological Center

8908 Guard St. E3831, Gunpowder, MD 21010

Email: nathan.d.mcdonald5.civ@army.mil

Keywords: Lipopolysaccharide, CRISPR-Cas9, Genome Engineering, Lipid A

***Yersinia pestis* 37C** ***Yersinia pestis* 26-28C**

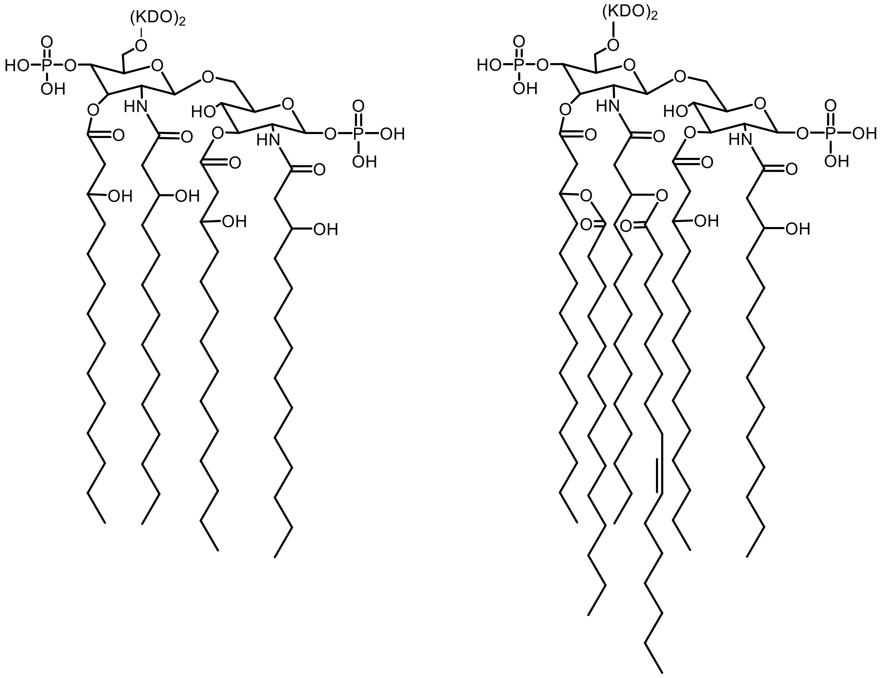

Supplementary Figure 1: *Yersinia pestis* lipid A structures at different temperatures. At lower temperatures, 26-28°C, the *Y. pestis* lipid A is hexa-acylated with the addition of a palmitoleate residue, catalyzed by the enzyme LpxP, and a myristate group catalyzed by the enzyme LpxM. As the temperature rises the lipid A is tetra-acylated.

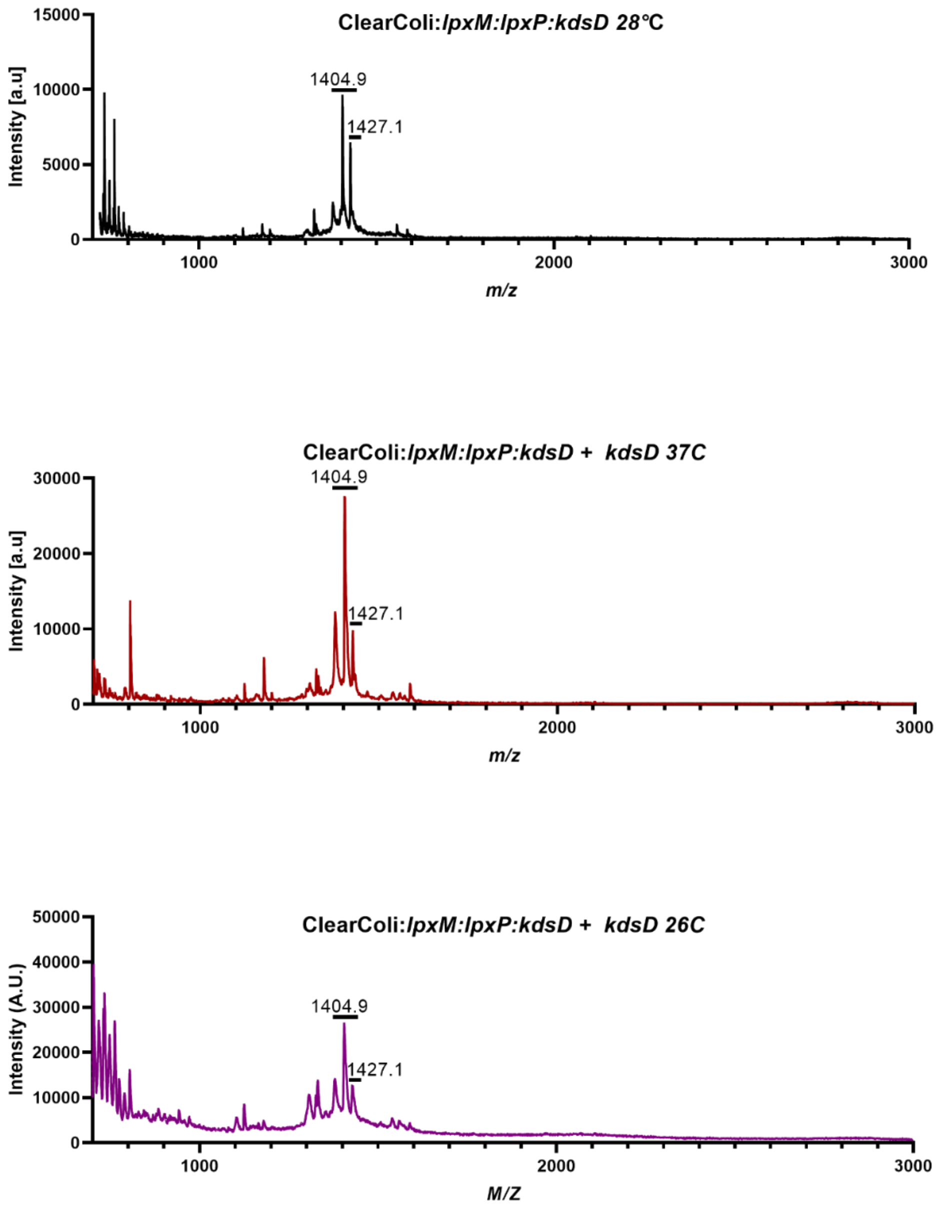

Supplemental Figure 2: MALDI-TOF characterization of lipid A structures isolated from engineered ClearColi strains. Lipids were isolated from A) ClearColi:*lpxM:lpxP:kdsD* which was grown at 28°C. The masses of 1405 *m/z* and 1427 *m/z* correspond to lipid IV_a and lipid IV_a+Na. B) Lipids isolated from ClearColi:*lpxM:lpxP:kdsD* + *kdsD* grown at 37°C or C) 26°C masses of 1405 *m/z* and 1427 *m/z* correspond to lipid IV_a and lipid IV_a+Na.

Sequence of inducible kdsD plasmid:

CCCGTAGAAAAGATCAAAGGATCTTCTTGAGATCCTTTTTTTCTGCGCGTAATCTGCTGCTTGCAAACAAAAAAACC
ACCGCTACCAGCGGTGGTTTGTTTGCCGGATCAAGAGCTACCAACTCTTTTTCCGAAGGTAACTGGCTTCAGCAGA
GCGCAGATACCAAATACTGTTCTTCTAGTGTAGCCGTAGTTAGCCCACCACTTCAAGAACTCTGTAGCACCGCCTAC
ATACCTCGCTCTGCTAATCCTGTTACCAGTGGCTGCTGCCAGTGGCGATAAGTCGTGTCTTACCGGGTTGGACTCA
AGACGATAGTTACCGGATAAGGCGCAGCGGTCGGGCTGAACGGGGGGTTCGTGCACACAGCCCAGCTTGGAGCG
AACGACCTACACCGAACTGAGATACCTACAGCGTGAGCTATGAGAAAGCGCCACGCTTCCCGAAGGGAGAAAGG
CGGACAGGTATCCGGTAAGCGGCAGGGTCGGAACAGGAGAGCGCACGAGGGAGCTTCCAGGGGGAAACGCCTG
GTATCTTTATAGTCCTGTCGGGTTTCGCCACCTCTGACTTGAGCGTCGATTTTTGTGATGCTCGTCAGGGGGGCGG
AGCCTATGGAAAAACGCCAGCAACGCGGCCTTTTTACGGTTCCTGGCCTTTTGCTGGCCTTTTGCTCACATGTTCTT
TCCTGCGTTATCCCCTGATTCTGTGGATAACCGTATTACCGCCTTTGAGTGAGCTGATACCGCTCGCCGCAGCCGAA
CGACCGAGCGCAGCGAGTCAGTGAGCGAGGAAGCGGAAGGCGAGAGTAGGGAACTGCCAGGCATCAAACTAAG
CAGAAGGCCCCTGACGGATGGCCTTTTTGCGTTTCTACAAACTCTTTCTGTGTTGTAAAACGACGGCCAGTCTTAAG
CTCGGGCCCCCTGGGCGGTTCTGATAACGAGTAATCGTTAATCCGCAAATAACGTAAAAACCCGCTTCGGCGGGTT
TTTTTATGGGGGGAGTTTAGGGAAAGAGCATTTGTCAGAATATTTAAGGGCGCCTGTCACTTTGCTTGATATATGA
GAATTATTTAACCTTATAAATGAGAAAAAAGCAACGCACTTTAAATAAGATACGTTGCTTTTTCGATTGATGAACAC
CTATAATTAAACTATTCATCTATTATTTATGATTTTTTGTATATACAATATTTCTAGTTTGTTAAAGAGAATTAAGAAA
ATAAATCTCGAAAATAATAAAGGGAAAATCAGTTTTTGATATCAAAATTATACATGTCAACGATAATACAAAATAT
AATACAAACTATAAGATGTTATCAGTATTTATTATGCATTTAGAATAAATTTTGTGTCGCCCTTCCGCGAAATTAATA
CGACTCACTATAGGGGAATTGTGAGCGGATAACAATTCCCCTCTAGAAATAATTTTGTTTAACTTTTTGAGACCTTA
ATTAGGTAAAAAATAAAAAATGTCTACTTTTGATTTACAGCCGGGCGTGGATTTCCAGCAGGCAGGTAAGCAGGT
ACTTCAGATTGAACGCGAAGGGCTGGCTCAACTCGATCAATACATAAACGAGGATTTCTCCAGAGCCTGTGAGGC
CATTTTTCGTTGCCACGGCAAAGTCGTCGTGATGGGAATGGGCAAATCTGGTCATATTGGCTGCAAAATTGCAGCC
ACATTCGCCAGCACGGGCACACCTGCATTTTTTGTCCATCCTGGTGAAGCCAGCCATGGCGATTTGGGCATGATCA
CGCCACAAGATATTGTATTAGCTATCTCTAACTCTGGGGAATCCAACGAAATCCTCACGTTGATCCCCGTGCTTAAG
CGCCAGAAAATTCTGTTGATTTGCATGAGCAGTAACCCTGAGAGCACCATGGGTAAAGCGGCCGATATTCACTTGT
GCATTAATGTGCCACAAGAGGCCTGTCCGCTGGGGCTAGCGCCAACCACCAGTACGACTGCAACACTGGTTATGG
GGGATGCGTTGGCGGTAGCCTTGCTCAAAGCACGGGGTTTCACGCAGGAAGATTTCGCACTCTCTCACCCAGGTG
GCGCGCTGGGGCGTAAGTTGCTGCTGCGGATCAGCGATATTATGCATACGGGTACGGAGATCCCCACCGTCAGCC
CTGATGCATCATTACGTGATGCCTTGCTAGAAATTACTCGGAAAAGTCTGGGTTTGACCGTTATTTGTGACGATTCA
ATGAGGATTAAAGGTATCTTTACCGACGGTGACTTGCGCCGGGTATTTGATATGGGCATTGATCTGAATAATGCAA
AAATTGCTGACGTCATGACTCGCGGGGGTATTCGAGTCCCTCCGAACATATTGGCGGTGGACGCGCTCAATCTGAT
GGAGTCACGCCATATCACTGCGTTGCTCGTCGCTGATGGTGACCAATTACTGGGTGTCGTACATATGCACGATATG
CTGAGAGCCGGTGTTGTCTGAGGTTGAGGTCTCACCCCCTAGCATAACCCCTTGGGGCCTCTAAACGGGTCTTGAG
GGGTTTTTTGCCCCTGAGACGCGTCAATCGAGTTCGTACCTAAGGGCGACACCCCCTAATTAGCCCGGGCGAAAG
GCCCAGTCTTTCGACTGAGCCTTTCGTTTTATTTGATGCCTGGCAGTTCCCTACTCTCGCATGGGGAGTCCCCACAC
TACCATCGGCGCTACGGCGTTTCACTTCTGAGTTCGGCATGGGGTCAGGTGGGACCACCGCGCTACTGCCGCCAG
GCAAACAAGGGGTGTTATGAGCCATATTCAGGTATAAATGGGCTCGCGATAATGTTCAGAATTGGTTAATTGGTT
GTAACACTGACCCCTATTTGTTTATTTTTCTAAATACATTCAAATATGTATCCGCTCATGAGACAATAACCCTGATAA
ATGCTTCAATAATATTGAAAAAGGAAGAATATGAGCCATATTCAACGGGAAACGTCGAGGCCGCGATTAAATTCC
AACATGGATGCTGATTTATATGGGTATAAATGGGCTCGCGATAATGTCGGGCAATCAGGTGCGACAATCTATCGC
TTGTATGGGAAGCCCGATGCGCCAGAGTTGTTTCTGAAACATGGCAAAGGTAGCGTTGCCAATGATGTTACAGAT
GAGATGGTCAGACTAAACTGGCTGACGGAATTTATGCCACTTCCGACCATCAAGCATTTTATCCGTACTCCTGATG
ATGCATGGTTACTCACCACTGCGATCCCCGGAAAAACAGCGTTCCAGGTATTAGAAGAATATCCTGATTCAGGTGA

AAATATTGTTGATGCGCTGGCAGTGTTCCTGCGCCGGTTGCACTCGATTCCTGTTTGTAATTGTCCTTTTAACAGCG
ATCGCGTATTTCGCCTCGCTCAGGCGCAATCACGAATGAATAACGGTTTGGTTGATGCGAGTGATTTTGATGACGA
GCGTAATGGCTGGCCTGTTGAACAAGTCTGGAAAGAAATGCATAAACTTTTGCCATTCTCACCGGATTCAGTCGTC
ACTCATGGTGATTTCTCACTTGATAACCTTATTTTTGACGAGGGGAAATTAATAGGTTGTATTGATGTTGGACGAGT
CGGAATCGCAGACCGATACCAGGATCTTGCCATCCTATGGAACTGCCTCGGTGAGTTTTCTCCTTCATTACAGAAA
CGGCTTTTTCAAAAATATGGTATTGATAATCCTGATATGAATAAATTGCAGTTTCATTTGATGCTCGATGAGTTTTTC
TAAGCGGCGCGCCATCGAATGGCGCAAAACCTTTCGCGGTATGGCATGATAGCGCCCGGAAGAGAGTCAATTCA
GGGTGGTGAATATGAAACCAGTAACGTTATACGATGTCGCAGAGTATGCCGGTGTCTCTTATCAGACCGTTTCCCG
CGTGGTGAACCAGGCCAGCCACGTTTCTGCGAAAACGCGGGAAAAAGTGGAAGCGGCGATGGCGGAGCTGAATT
ACATTCCCAACCGCGTGGCACAACAACTGGCGGGCAAACAGTCGTTGCTGATTGGCGTTGCCACCTCCAGTCTGGC
CCTGCACGCGCCGTCGCAAATTGTCGCGGCGATTAAATCTCGCGCCGATCAACTGGGTGCCAGCGTGGTGGTGTC
GATGGTAGAACGAAGCGGCGTCGAAGCCTGTAAAGCGGCGGTGCACAATCTTCTCGCGCAACGCGTCAGTGGGC
TGATCATTAACTATCCGCTGGATGACCAGGATGCCATTGCTGTGGAAGCTGCCTGCACTAATGTTCCGGCGTTATTT
CTTGATGTCTCTGACCAGACACCCATCAACAGTATTATTTTCTCCCATGAGGACGGTACGCGACTGGGCGTGGAGC
ATCTGGTCGCATTGGGTCACCAGCAAATCGCGCTGTTAGCGGGCCCATTAAGTTCTGTCTCGGCGCGTCTGCGTCT
GGCTGGCTGGCATAAATATCTCACTCGCAATCAAATTCAGCCGATAGCGGAACGGGAAGGCGACTGGAGTGCCAT
GTCCGGTTTTCAACAAACCATGCAAATGCTGAATGAGGGCATCGTTCCCACTGCGATGCTGGTTGCCAACGATCAG
ATGGCGCTGGGCGCAATGCGCGCCATTACCGAGTCCGGGCTGCGCGTTGGTGCGGATATCTCGGTAGTGGGATA
CGACGATACCGAAGATAGCTCATGTTATATCCCGCCGTTAACCACCATCAAACAGGATTTTCGCCTGCTGGGGCAA
ACCAGCGTGGACCGCTTGCTGCAACTCTCTCAGGGCCAGGCGGTGAAGGGCAATCAGCTGTTGCCAGTCTCACTG
GTGAAAAGAAAAACCACCCTGGCGCCCAATACGCAAACCGCCTCTCCCCGCGCGTTGGCCGATTCATTAATGCAGC
TGGCACGACAGGTTTCCCGACTGGAAAGCGGGCAGTGACTCATGACCAAAATCCCTTAACGTGAGTTACGCGCGC
GTCGTTCCACTGAGCGTCAGAC

T7 inducible promotor

kdsD coding sequence

T7_term

Repair templates ordered as gBlocks

E.coli gutQ locus *Y. pestis kdsD* gene

gcgactcaataattctggatgctggcagtaccgttttgcagatggtgcccctgctctcgcgctttaataacatcacggtgatgaccaacagcctgcatat
cgtcaatgcgctatccgaactggataacgaacaaactatcctgatgccaggcggaacatttcgtaaaaaatcggcctcatttcacgggcagctggcag
agaatgccttcgagcatttcaccttcgataaattgtttatgggcaccgacggcatcgatctcaatgcgggcgtaaccacctttaacgaggtttataccgtc
agtaaggcaatgtgcaatgccgcgcgcgaagtgattttgatggcggactcatcaaagtttggccgtaaaagccccaacgtagtttgcagtcttgaaagc
gtcgataagctgattaccgacgcaggtatcgatccggcgtttcgtcaggcgctggaagagaaagggatcgatgtgatcataaccggagagagcaatg
agtgaagcgtgtaggctggagctgcttcgaagttcctatactttctagagaataggaacttcggaataggaactaagg**agg**tcagacaacaccggctc
tcagcatatcgtgcatatgtacgacacccagtaattggtcaccatcagcgacgagcaacgcagtgatatggcgtgactccatcagattgagcgcgtcc
accgccaatatgttcggagggactcgaatacccccgcgagtcatgacgtcagcaatttttgcattattcagatcaatgcccatatcaaatacccggcgc
aagtcaccgtcggtaaagatacctttaatcctcattgaatcgtcacaaataacggtcaaacccagacttttccgagtaatttctagcaaggcatcacgta
atgatgcatcagggctgacggtggggatctccgtacccgtatgcataatatcgctgatccgcagcagcaacttacgccccagcgcgccacctgggtga
gagagtgcgaaatcttcctgcgtgaaaccccgtgctttgagcaaggctaccgccaacgcatcccccataaccagtgttgcagtcgtactggtggttggc

gctagccccagcggacaggcctcttgtggcacattaatgcacaagtgaatatcggccgctttacccatggtgctctcagggttactgctcatgcaaatca
acagaattttctggcgcttaagcacggggatcaacgtgaggatttcgttggattccccagagttagagatagctaatacaatatcttgtggcgtgatcat
gcccaaatcgccatggctggcttcaccaggatggacaaaaaatgcaggtgtgcccgtgctggcgaatgtggctgcaattttgcagccaatatgaccag
atttgcccattcccatcacgacgactttgccgtggcaacgaaaaatggcctcacaggctctggagaaatcctcgtttatgtattgatcgagttgagccag
cccttcgcgttcaatctgaagtacctgcttacctgcctgctggaaatccacgcccggctgtaaatcaaaagtagacatatattcatatggattatttaatc
cttcaatcccagacgtttcgccagccgatgcaggttggcgacgtcggtttccagcatccgcgcgcaggcagcccagttgtgatgattttgtgccagtgcc
tgacgaatagtttcacgctggaacgcttctgtcgcttcacgcaggttttgcttaacaacgggcaccgccgccacttctggcgtcggcaacgtcacctcag
gaaaagcaaaatgttgcgcctcaagaatcacttcatcgccgctgcgggtggctctcgccagaactaccgcccgatgaatagcatgttccagttcgcgca
cgtttcccggaaaactgtagtgttgcagtaaatttcgcgctccggcacttaataccacgcgggagagcccctgccgcaaacgacactgctcgcagaaat
accccgccagcagaatgacatcatcgccccgctcacgcagcggcggcaccgaaagtggaaacacgctcaggcgatgaaacaaatcggcg

Yellow highlighted region: Clearcoli genome homology
Bold: PAM
Turquoise: *Y. pestis kdsD* gene

E.coli lpxM locus *Y. pestis lpxM* gene

cgagtcggtgctttttttgagtggcgttacgccggtgcactttgatagcgctaatgacggcgtagcagctgccagcgaagcggttaatctgctgcgcgat
aaaggttacttgatgtctggtgacctggtgattgtcacccagggcgacgtgatgagtaccgtgggttctactaataccacgcgtattttaacggtagagt
aagtacgttgccggatgcggcgaaaacgccacatccggcctacagttcaatgatagttcaacagatttcgaatattctgaagcaaacttgaacttatca
tcaggcgaaggcctctcctcgcgagaggcttttaatatcctccttagttcctattccgaagttcctattctctagaaagaataggaacttcgaagcagctc
cagccaattgacctaaagatctttgcgcacatagggctcggtttcaccctctttccgggtttttaacagctttaagatccatgtatattgctctggattcggc
ttaaccaacaactccacttcctcattcatccggcgggcaatataggcgtcatcagcatccgccaaatcatccattggcggacggatatagatatcaaga
cgatgctctctataattataaacagggaacatcggtacaatggccgcacggcagactttcattaaacggcctattgccggtaatgtcgccttataggtcg
cgaagaaatcaacaaattcactttgctcaggaccataatcttcatcaggtaagtagtaaccccagaatccttggcgtactgaactgataaaggtttga
tgccactttcgcgggcatggatccgaccaccgaaatgtaaacgcgcactgttccatagatagtccaccaatggattacgttgatgatggaacatacctg
caactggcttgccctgctccgctagcagcatagccggaatatcaatggaccaggcatgggggaccaacaaaataacattacgctcttgttgttgcagct
catccagtatttcctgtccatgccagtgcacgcgagtcagcactttttcggatcacgaaaacaaagttctgccatcatcattaacggttgagctgcggta
gcaaacatctggtcaataatgtgttcgcgttcagactcgggtaactcaggcatacaataaagaagattaatacgagcgcgacgacgggcgcttttagc
aaatttcccagcgagacgccctatgccagctaataaaggatcacggaatttgggtggaatataagcgagcgccgcaatcgctccagcacctaaccaa
accccccagaaacgcggatggaggaaggttttctgaaataccggaataaaaccagttgcgtcatttttttctttgtgcat**cgg**tcgcttttccagtttcgg
ataaggcaaaaatcaatctggtgatagtgtagcggcgcaacttgccccgcaccaaataaaaaaagccggtactgactgcgtaccggctgcgaatgga
tgttaattaatcaaaccgtagctgcggcacaatctctttggcctgcgccaggaattcgcgacgatcggagccggtcagcccttcggtacgcggcagtttt
gccgtcagcgggtttacggcctgctggtttatccatacttcatagtgcagatgcggcccggttgaacgtccggtattaccggaaagcgcgatacggtcgc
cacgtttcaccttctgtcccggtttcaccagaatcttgcgcaagtgcatataacgcgtggtgtagctgcgaccatgaattctctagagtcgacctgc
Yellow highlighted region: Clearcoli genome homology
Bold: PAM
Turquoise: *Y. pestis lpxM* gene

E.coli lpxP locus *Y. pestis lpxP* gene

aacatccataaataaatcatagctcaacctcctgccgcgctaaaaaacctgtaacttcagtcactttaatagtaagacaatccttaacctctggcaattt
ttgctaagcatcaatgaaaacagcatgttaaatgcaagactgttgtgtacggaaaaatatttactttgcacgattaataatcagtagctgaaagcagtc
agcgagtgataaagtacagataccgcattcatgctcctcgtggttatgtcctgactagtctttacactctttacaggaaccattgtcgtacatgatggccc
aaccaattgagtgtaccgctgtcgacatggaatcttctgcgatacaacaattcgtatctacagaaggtaactgtgtttaatacagcgaaggttcgccaat
gggtcgggttttaaagcggcggtgtaaccaaagatattgttccggcgcgcgcatgatctctttctcaatcactttattcatatagctggcagcggctatct
catcgtcaatcgggtagtcttccagcgcgggttggatcagcaggtcatagccacggccctctttttgcgcaataaaaccaagggcagcagtgcgggttt

tgctaaacgggcgagcataaaggtgccgctggtggttgccgcatgttctacggcgaacagtggtgcaaaaacgctgccgcgcgggccataatcttgat
ctggtgcaaaccataccgcttcaccctgcttcaaggctcgcaccataccgcgcaggtctctccgatccaacatcgctttgttggagcgcatacggcccca
ggtctgcactaattccatcactttattgttgtgtgggcgatacatcgccatcattggctgacattgccccatgacccggccacccagctctaatgacataa
aatggacgccaatgaccagtacgccccgtttaccttgttgtgcgcgtttcaggttctccagaccggagacggaaaaccagcgctgtatccgggtatcgg
accaaaaccacgccatcccggtttccagcaatcccatgcccaatgattcgaaattaccgatgatagtctgctcaaggacttgtttatccatgtcaggaaa
acagagttcaagattacgtcgggcaatggaaacccgccgtttgaggaaccgcatggaggttcgccccaaccaaacacctaacttattgagtagtgggt
aagggagttgaactaacaaaaacagtacgccaaggccaaaccaggttagccaatagcgtgggtgtagcagggaaatatggaatttctgcggctttat
cat**agg**ctggagctgcttcgaagttcctatactttctagagaataggaacttcgtgaataggaactaaggaggatattcatatgcaaactgctcttactc
tttccctcctcaaatcggccataataacctcatggtttttatggctttttcttaaggtttttccttcgctgaatatctaattattaccgtcagaaattaaactg
tcgccgatatgagacacttgtaagttactaaaagtaaataagaaaatgcctcttgtctcgtttcagttttaggcgtacattagcgccgtctcgagcagttt
agctcagaattctggggtgatgcccacggtaaaatacagggcttttaaggtcgctttgcccggtgtcaactcactgtatcaggtgtaatgaagtcattca
ggcgtaacagtaattacgcggagagatgtaaagtgaaatatttctttat

Yellow highlighted region: Clearcoli genome homology
Bold: PAM
Turquoise: Y. pestis *lpxP* gene
